# Telomere Shortening Drives Atrial Fibrillation Through VCAM‐1 Mediated Atrial Electrical and Structural Remodeling

**DOI:** 10.1111/acel.70417

**Published:** 2026-02-13

**Authors:** Zhaojia Wang, Rui Zhao, Yuwen Wang, Nan Zhang, Qiuhui Yang, Zandong Zhou, Duo Jiang, Xu Zhang, Jinghua Yuan, Yi Zheng, Wenhua Song, Daiqi Liu, Xunzhi Liu, Kejing Yuan, Gary Tse, Gregory Y. H. Lip, Tong Liu, Feng Wang

**Affiliations:** ^1^ Tianjin Key Laboratory of Ion and Molecular Function of Cardiovascular Diseases, Department of Cardiology Second Hospital of Tianjin Medical University, Tianjin Institute of Cardiology Tianjin China; ^2^ Department of Genetics, School of Basic Medical Science Tianjin Medical University, The Province and Ministry Co‐Sponsored Collaborative Innovation Center for Medical Epigenetics Tianjin People's Republic of China; ^3^ Key Laboratory of Artificial Organs and Computational Medicine in Zhejiang Province Shulan International Medical College, Zhejiang Shuren University Hangzhou People's Republic of China; ^4^ Shanghai Fuyang Biotechnology Co., LTD Shanghai China; ^5^ School of Nursing and Health Sciences Hong Kong Metropolitan University Hong Kong China; ^6^ Liverpool Centre for Cardiovascular Science at University of Liverpool, Liverpool John Moores University and Liverpool Heart & Chest Hospital Liverpool UK; ^7^ Department of Clinical Medicine Aalborg University Aalborg Denmark; ^8^ Medical University of Bialystok Bialystok Poland; ^9^ Tianjin Key Laboratory of Cellular and Molecular Immunology Tianjin People's Republic of China

**Keywords:** aging, atrial fibrillation, telomere, *TERT*, VCAM‐1

## Abstract

Telomere shortening is a hallmark of aging and has been implicated in cardiovascular disease, but its mechanistic link to atrial fibrillation (AF) remains elusive. Using a high‐throughput, single‐gene‐calibrated dot blot assay, we developed to quantify leukocyte telomere length (LTL). In age‐stratified analyses, shorter LTL was associated with AF predominantly in individuals younger than 70 years. In telomerase‐deficient (*TERT* ^−/−^) mice with telomere dysfunction, higher AF inducibility, atrial electrical conduction slowing, and atrial fibrosis were observed. Transcriptomic profiling revealed significant alterations in extracellular matrix and cell adhesion pathways in response to telomere dysfunction. Subsequent validation identified vascular cell adhesion molecule‐1 (VCAM‐1) as a potential mediator linking telomere shortening to AF‐related atrial remodeling. Functional inhibition of VCAM‐1 reversed electrophysiological abnormalities, attenuated atrial fibrosis, normalized ECM gene expression—including Col1α1, α‐SMA, and CD168—and reduced AF susceptibility by 30%. These findings establish a telomere–VCAM‐1 axis that drives atrial remodeling and arrhythmogenesis in aging, and position VCAM‐1 as a candidate therapeutic target for age‐related AF.

## Graphical abstract

This study reveals that telomere shortening promotes atrial fibrillation by upregulating VCAM‐1, leading to fibrosis and electrical remodeling. Inhibiting VCAM‐1 reverses these abnormalities, identifying it as a therapeutic target for age‐related AF.

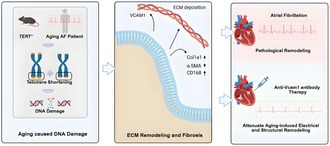

## 1 Introduction

Atrial fibrillation (AF) represents one of the most common persistent arrhythmias in clinical practice and is linked to substantial morbidity, notably an elevated risk of stroke, heart failure, and all‐cause mortality (Lip et al. 2019; Lippi et al. 2021). Its prevalence increases markedly with advancing age, rendering it a major clinical problem in aging populations. Epidemiological studies have shown that the risk of developing AF approximately doubles with every decade, resulting in a striking prevalence of 10%–20% in individuals over 80 years of age (Lloyd‐Jones et al. 2004; Heeringa et al. 2006; Magnani et al. 2016; Kornej et al. 2020). Despite its high prevalence and clinical burden, the pathophysiological mechanisms underlying AF remain incompletely understood. A thorough investigation into the complex interplay between aging and AF is essential for advancing targeted therapeutic approaches.

Telomeres, the protective caps located at the ends of chromosomes, play a pivotal role in cellular aging and replicative capacity (Bernadotte et al. 2016). Telomere shortening is a hallmark of aging and a key factor contributing to age‐related cardiovascular disorders (Tribulova et al. 2015; Chiriacò et al. 2019; Ale‐Agha et al. 2021; Brandt et al. 2022; Schuermans et al. 2023). However, investigations into the association between leukocyte telomere length (LTL) and AF in elderly populations have yielded inconsistent findings (Roberts et al. 2014; Staerk et al. 2017; Zhang et al. 2018; Liu et al. 2022; Zheng et al. 2022). The lack of consensus highlights the pressing need to elucidate the mechanistic link between telomere dysfunction and AF pathogenesis.

We also explore the role of vascular cell adhesion molecule‐1 (VCAM‐1) as a potential mediator linking telomere dysfunction to AF. VCAM‐1, a cell adhesion molecule, is frequently upregulated in aged tissues and has been implicated in inflammatory and fibrotic responses in cardiovascular diseases (Yin et al. 2022; Ma et al. 2023) (Qiu et al. 2022). Although previous studies have linked elevated VCAM‐1 levels to atrial fibrosis and impaired atrial function (Kume et al. 2017; Mathew et al. 2024), its specific role in telomere dysfunction‐induced AF has not been fully elucidated. Here, our research will explore the mechanistic role of VCAM‐1 in driving atrial remodeling and AF in the setting of telomere attrition.

In this study, we set out to test the hypothesis that telomere shortening, as a marker of biological aging, contributes to atrial fibrillation (AF) susceptibility by promoting atrial electrical and structural remodeling through specific molecular mediators. To address this, we first developed a high‐throughput, single‐gene–calibrated dot blot method for accurate measurement of leukocyte telomere length (LTL), and applied this approach to a clinical cohort. And both the clinical cohort and UK Biobank population to examine the association between LTL and AF in patients across different age groups. We then employed a telomerase‐deficient (*TERT* ^−/−^) mouse model to investigate the impact of telomere dysfunction on atrial electrophysiology and structural remodeling. Transcriptomic profiling was used to identify candidate pathways linking telomere dysfunction to atrial remodeling, followed by targeted functional intervention to interrogate the role of vascular cell adhesion molecule‐1 (VCAM‐1). Together, this integrated experimental and population‐based approach was designed to elucidate mechanistic links between telomere attrition, atrial remodeling, and AF, independent of advanced chronological age, without presupposing specific outcomes.

## 2 Results

### 2.1 Age‐Dependent Correlation Between Leukocyte Telomere Length and Atrial Fibrillation as Revealed by High‐Throughput Dot Blot Hybridization

The relationships between aging, LTL, and the development of AF have been examined in previous studies (Iwasaki et al. 2011; Zheng et al. 2022). In these studies, telomere length was typically assessed using PCR‐based techniques. While PCR offers high throughput and operational efficiency, it suffers from limited accuracy due to amplification bias and telomeric sequence complexity. To overcome these limitations, we developed a novel dot blot hybridization method calibrated with a single‐copy gene, designed specifically for high‐throughput detection in population‐scale samples. Compared with conventional approaches such as terminal restriction fragment (TRF) analysis and PCR, our technique provides enhanced accuracy, scalability, procedural simplicity, and cost‐effectiveness.

**FIGURE 1:**
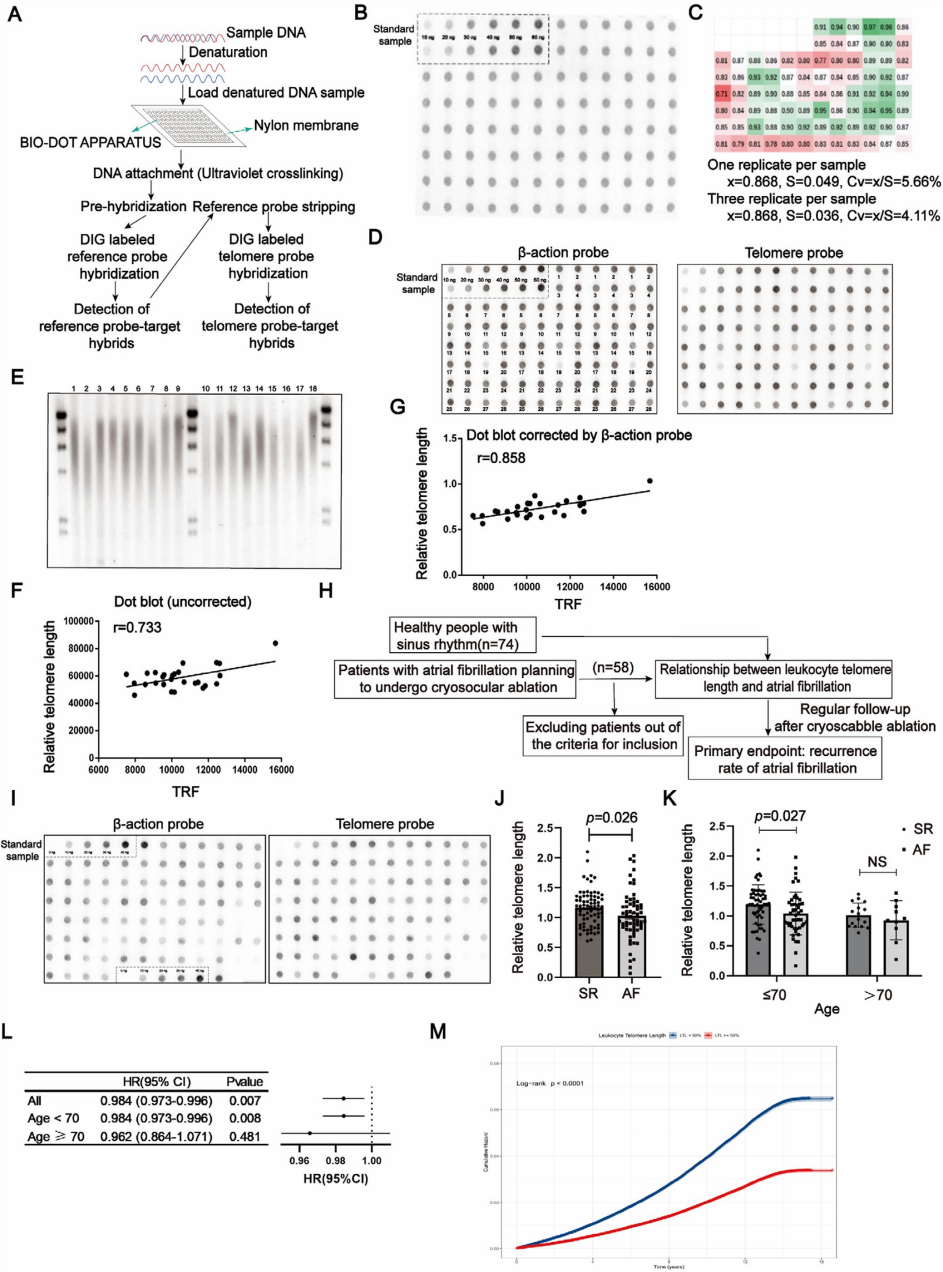
High‐throughput dot blot hybridization analysis of leukocyte telomere length in atrial fibrillation and sinus rhythm control populations. (A) Schematic representation of the quantitative dot blot workflow used to measure telomere length in human peripheral blood DNA. (B) Dot blot analysis of telomere length in human peripheral blood DNA. The dashed box indicates the standard DNA samples (10, 20, 30, 40, 50, 60 ng), while the remaining spots correspond to identical DNA samples. (C) The relative telomere lengths for 84 spots from the same sample were calculated, resulting in a coefficient of variation (CV) of 5.66%. Averaging the results from triplicate spots yielded a CV of 4.11% among the 28 calculated telomere lengths. (D) Dot blot analysis of telomere length in human peripheral blood DNA, with β‐actin serving as the reference probe for normalization. Sample IDs (*n* = 28) are marked in the image, with each sample run in triplicate. (E) Measurement of telomere length in human peripheral blood DNA using terminal restriction fragment (TRF) analysis. The same samples as those in panel C were analyzed. (F, G) Correlation analysis between telomere length measurements obtained from dot blot and TRF methods, both before (F) and after correction (G) with the reference probe (*n* = 28). (H) Flowchart illustrating the patient selection process for the study, highlighting the inclusion of patients with atrial fibrillation (AF) and sinus rhythm controls. (I) Dot blot analysis showing relative leukocyte telomere length in individuals with sinus rhythm compared to those with AF. (J) Comparison of relative leukocyte telomere length between sinus rhythm group and AF patient group. (K) Analysis of relative leukocyte telomere length across different age groups, comparing individuals with sinus rhythm and AF. Statistical significance was assessed at *p* < 0.05, and data are presented as mean ± SEM. (L) Association Between Leukocyte Telomere Length (LTL) and Incident Atrial Fibrillation/Flutter. All models were adjusted for age, sex, race, smoking status, BMI, systolic blood pressure, diastolic blood pressure, antihypertensive medication, and diabetes mellitus. (M) Multivariable‐adjusted Kaplan–Meier Curves Depicting the Cumulative Risk of Atrial Fibrillation Across Two Groups Defined by Mean LTL. The cumulative risk estimates were adjusted for age, sex, race, smoking status, BMI, systolic blood pressure, diastolic blood pressure, antihypertensive medication, and diabetes mellitus.

The technique is grounded in a denatured‐Southern Blot hybridization platform to achieve precise telomere length determination (Figure 1A). Thirty nanograms of genomic DNA were applied per well, followed by hybridization with both a telomere‐specific probe and a DIG‐labeled β‐actin probe, the latter serving as an internal reference to normalize telomere signal intensity (Figure 1B). The assay demonstrated strong reproducibility, with a batch‐to‐batch coefficient of variation (CV) of 5.66%. When samples were processed in duplicate or triplicate, CVs were further reduced to 4.25% and 4.11%, respectively, indicating high technical precision (Figure 1C). For subsequent analyses, all samples were run in duplicate and averaged to enhance consistency. Initial benchmarking against the gold‐standard TRF method revealed a significant correlation (*r* = 0.733, *p* = 0.032) (Figure 1D–F). Following normalization using the β‐actin probe—applied after telomere probe stripping—the correlation improved to *r* = 0.858 (*p* = 0.027), reflecting enhanced accuracy and control over sample loading variability (Figure 1G). Moreover, the protocol is compatible with both 96‐ and 384‐well formats, enabling rapid, high‐throughput processing of large sample cohorts, making it ideally suited for population‐scale and clinical applications.

Using this platform, we measured telomere length in 74 healthy individuals with normal sinus rhythm and 58 patients with atrial fibrillation (AF). Clinical characteristics of the participants are shown in Tables 1 and 2. The results revealed that the AF group exhibited significantly shorter leukocyte telomere lengths (LTLs) overall compared to controls (Figure 1H–J) (*p* = 0.026). Additionally, age‐stratified analysis revealed that in individuals under 70 years of age, shorter LTLs correlated with increased AF prevalence, whereas this association was attenuated in individuals aged 70 and above (*p* > 0.05) (Figure 1K). These findings suggest that shortened LTL is a prominent risk factor for AF in young‐old populations (median age 63 years; interquartile range 57–67), while other age‐related pathophysiological factors may overshadow this association in advanced age. Furthermore, we next compared LTLs among individuals with recurrent AF, non‐recurrent AF (Figure S1A), but we didn't observe any significant difference between these two groups.

**TABLE 1:** Clinical characteristics of the participants.

Characteristics	Healthy individuals with sinus rhythm (SR) (*n* = 74)	Patients with atrial fibrillation (AF) (*n* = 46)
Female (%)	25 (33.78)	26 (44.83)
Age (years)	63.00 (57.75, 69.00)	63.00 (57.00, 67.75)
BMI (kg/m^2^)	23.60 (21.13, 27.80)	26.25 (24.65, 28.15)
Systolic blood pressure (mmHg)	136.50 (116.75, 157.25)	130.00 (120.00, 139.00)
Diastolic blood pressure (mmHg)	88.50 (76.75, 97.25)	80.00 (74.25, 84.75)
HR (bpm)	78.50 (69.75, 90.25)	73.50 (65.25, 90.75)

**TABLE 2:** Clinical characteristics of the AF patients.

Characteristics	Total (*n* = 58)	Young‐old (*n* = 46)	Old‐old (*n* = 12)
Female (%)	26 (44.83)	17 (36.96)	9 (75.00)
Age (years)	63.00 (57.00, 67.75)	61.00 (54.25, 65.00)	73.50 (71.75, 77.25)
BMI (kg/m^2^)	26.25 (24.65, 28.15)	26.28 (24.78, 27.98)	25.53 (23.46, 29.54)
Paroxysmal atrial fibrillation (%)	42 (72.41)	35 (76.09)	7 (58.33)
NYHA class			
I (%)	19 (32.76)	18 (39.13)	1 (8.33)
II (%)	35 (60.34)	25 (54.35)	10 (83.33)
III (%)	4 (6.90)	3 (6.52)	1 (8.33)
IV (%)	0 (0.00)	0 (0.00)	0 (0.00)
Time of hospitalization (days)	4.00 (3.00, 5.75)	4.00 (3.00, 6.00)	4.00 (3.00, 5.00)
Systolic blood pressure (mmHg)	130.00 (120.00, 139.00)	129.50 (119.25, 138.75)	130.00 (121.00, 139.25)
Diastolic blood pressure (mmHg)	80.00 (74.25, 84.75)	81.00 (75.25, 85.00)	77.00 (70.50, 80.00)
HR (bpm)	73.50 (65.25, 90.75)	74.00 (64.25, 89.75)	72.00 (67.50, 91.75)
LAD (mm)	42.20 (38.20, 45.20)	42.20 (37.70, 45.10)	43.30 (40.30, 45.50)
LVEDD (mm)	47.80 (45.40, 51.90)	47.90 (45.50, 51.90)	46.95 (43.40, 50.33)
LVEF (%)	62.00 (59.00, 65.00)	63.00 (59.00, 65.00)	60.00 (56.75, 62.50)
RAD (mm)	37.50 (34.50, 43.10)	36.00 (34.30, 39.50)	41.05 (39.70, 45.43)
Potassium (mmol/L)	4.20 (3.88, 4.40)	4.20 (4.00, 4.40)	4.10 (3.73, 4.30)
Creatinine (μmol/L)	73.60 (63.25, 82.35)	71.50 (63.23, 81.43)	74.20 (64.20, 84.80)
LDL (mmol/L)	2.44 (2.20, 3.09)	2.47 (2.25, 3.18)	2.31 (2.13, 2.58)
Creatine kinase (U/L)	85.00 (59.50, 108.70)	85.00 (60.70, 117.05)	81.75 (53.18, 96.23)
Creatine kinase‐MB (U/L)	10.40 (8.24, 12.30)	10.40 (7.50, 12.20)	10.34 (9.85, 15.26)
NT‐pro BNP (ng/L)	848.80 (100.00, 1446.95)	840.00 (100.00, 1021.80)	1363.80 (345.55, 2341.70)
Concomitant disease			
Hypertension (%)	37 (63.79)	25 (54.35)	12 (100.00)
Diabetes mellitus (%)	19 (32.76)	16 (34.78)	3 (25.00)
Heart failure (%)	15 (25.86)	11 (23.91)	4 (33.33)
Medications			
Metoprolol (%)	53 (91.38)	43 (93.48)	10 (83.33)
Amiodarone (%)	5 (8.62)	5 (10.87)	0 (0.00)
Rivaroxaban (%)	12 (20.69)	10 (21.74)	2 (16.67)
Warfarin (%)	22 (37.93)	18 (39.13)	4 (33.33)

Abbreviations: BMI, body mass index; HR, heart rate; LAD, left atrial diameter; LDL, low‐density lipoprotein; LVEDD, left ventricular end‐diastolic dimension; LVEF, left ventricular ejection fraction; NT‐pro BNP, N‐terminal pro‐B type natriuretic peptide; NYHA, New York Heart Association; RAD, right atrium diameter.

To validate these findings at the population level, we performed a parallel analysis using the UK Biobank cohort, a large population‐based cohort study that recruited 500,000 individuals aged 37–73 years from across the UK between 2006 and 2010. During a mean follow‐up period of 15.47 years, 29,796 AF cases were recorded. In the Cox proportional hazards analysis, longer LTL was associated with a lower risk of AF (HR = 0.984/SD, 95% CI, 0.973–0.996, *p* = 0.007) (Figure 1L). Figure 1M presents the multivariable‐adjusted cumulative risk of AF based on the mean LTL values. Participants with shorter LTL had a higher probability of developing AF during follow‐up compared to those with longer LTL. We also observed evidence of an interaction between LTL and age (*p* = 0.007). In the stratified analysis, among participants younger than 70 years, an increase of 1 SD in LTL was associated with a hazard ratio (HR) of 0.984 (95% CI, 0.973–0.996; *p* = 0.008). However, in those aged 70 or older, no statistically significant association between longer LTL and AF events was observed (*p* = 0.481). These findings further supported the role of telomere shortening in AF development (Figure 1L,M).

### 2.2 Telomere Shortening Is Linked to Atrial Fibrillation Susceptibility in Mice Models

**FIGURE 2:**
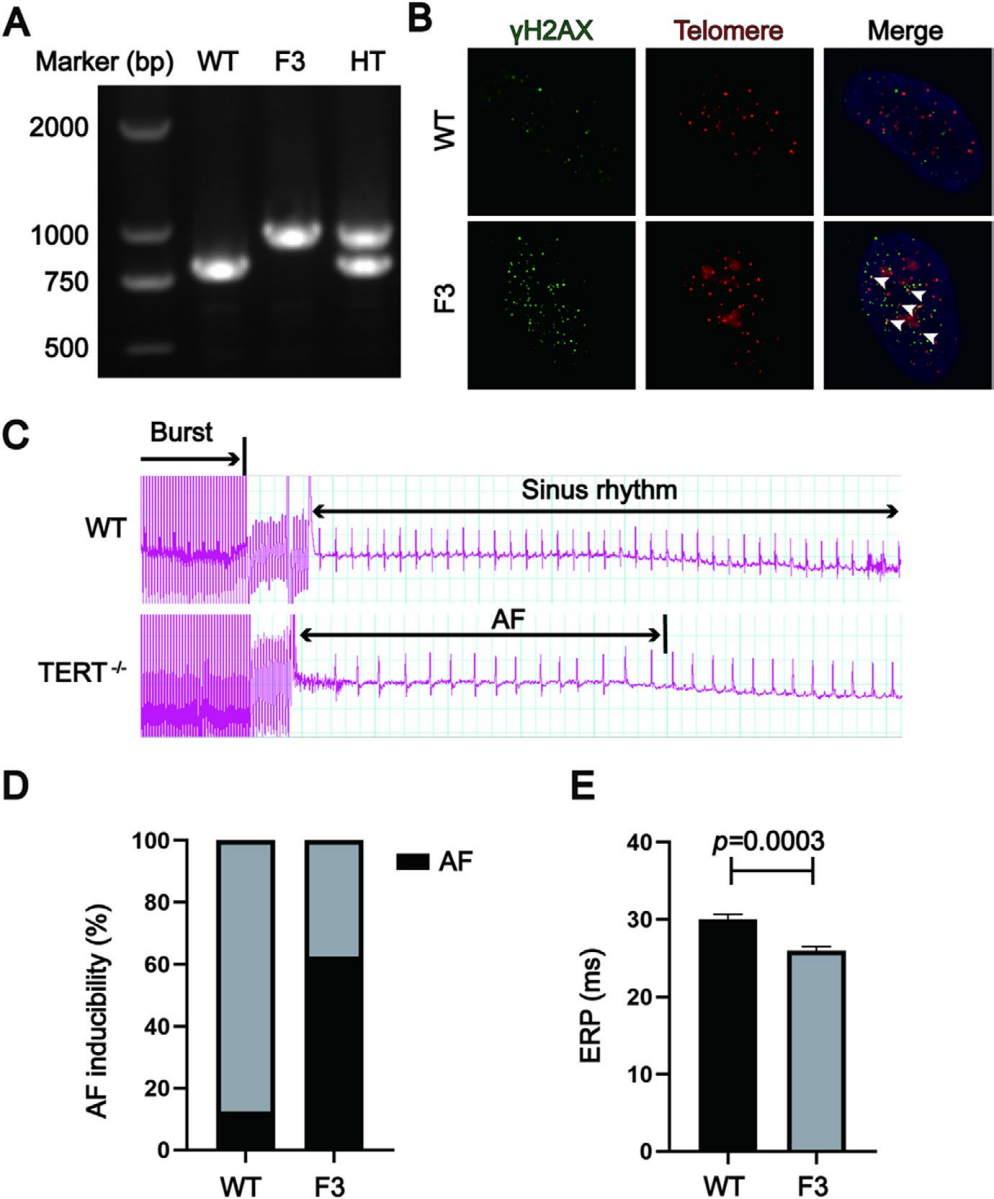
Telomere shortening and damage promote atrial fibrillation in mice. (A) PCR confirmation of genotype in *TERT* ^+/+^ (WT), *TERT* ^+/−^ (HT), and *TERT* ^−/−^ (F3) mice. WT mice exhibit a single band at 800 bp, F3 mice show a distinct band at 1000 bp, and HT mice display both 800 and 1000 bp bands, indicating heterozygosity. (B) Telomere damage assessment through the co‐localization of telomeres (red) and γH2AX (green) in bone marrow cells from WT and F3 mice, highlighting increased telomere‐associated DNA damage in F3 mice. (C–E) Analysis of AF inducibility in WT and F3 mice, with data showing a significant increase in AF susceptibility in F3 mice compared to WT. Sample size is *n* = 8, with statistical significance denoted by *p < 0.05. Data are presented as mean ± SEM.

To further investigate the correlation between telomere dysfunction and AF inducibility in mouse models, we constructed a telomerase reverse transcriptase knockout (*TERT* ^−/−^) mouse model (Figure 2A). In this model, the telomere length in WT mice was generally maintained at normal levels, whereas the third generation of *TERT* ^−/−^ mice (F3 mice) displayed significantly shortened telomeres (Figure S2A,B). Immunofluorescence analysis confirmed increased DNA damage foci, as indicated by elevated γH2AX levels (Figure 2B), validating the telomere damage phenotype.

Subsequently, an electrophysiological study was conducted using the WT and F3 mouse model to investigate the relationship between telomere dysfunction and its impact on AF inducibility. AF was induced by rapid pacing at the right atrium, and AF episodes were monitored using electrocardiograms (Figure 2C). A significant increase in AF inducibility rate was observed in the F3 mice compared to the WT group (62.5% vs. 12.5% after three inductions) (Figure 2D). This increase was accompanied by shortened atrial effective refractory periods (ERPs) (26 vs. 30 ms; *n* = 8) (Figure 2E). These findings indicate that telomere shortening increases AF inducibility by facilitating reentry‐based arrhythmic substrates.

### 2.3 Atrial Electrical Remodeling in *TERT* ^−/−^ Mice

**FIGURE 3:**
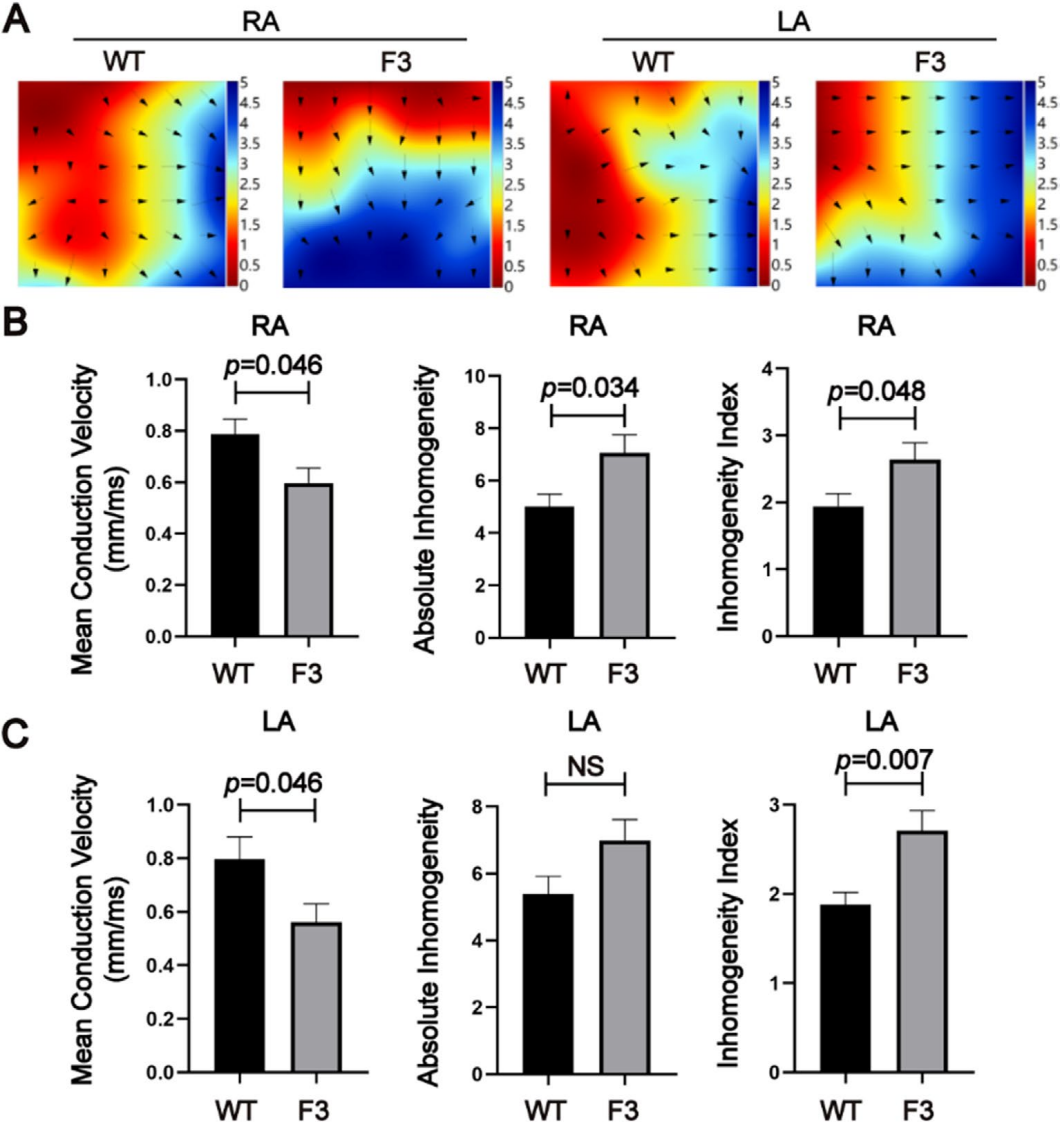
Telomere shortening in mice leads to atrial electrical conduction abnormalities. (A) Color‐coded simulated epicardial activation maps of the atrium in WT and F3 mice demonstrating alterations in electrical conduction patterns associated with telomere shortening. (B, C) Quantitative comparison of mean epicardial electrical conduction velocity, absolute inhomogeneity, and the inhomogeneity index of atrial conduction in WT and F3 mice, highlighting significant differences in conduction properties between the two groups. Data are presented as mean ± SEM (*n* = 6).

Atrial electrical remodeling is crucial for promoting the development of AF. To assess electrophysiological remodeling, we performed electrical mapping of both atria (Figure 3A). Our analysis revealed that F3 mice showed a significant reduction in mean epicardial electrical conduction velocity in the right atrium compared to the WT mice. Additionally, there were increased levels of absolute inhomogeneity and a higher inhomogeneity index (Figure 3B). Similar patterns were observed in the left atrium, where F3 mice had decreased conduction velocity and elevated conduction absolute heterogeneity, along with a higher inhomogeneity index (Figure 3C). Although differences in absolute inhomogeneity did not reach full statistical significance, a clear trend was evident. These findings indicate that telomere dysfunction impairs atrial electrical stability, characterized by slowed and heterogeneous conduction.

### 2.4 VCAM‐1 Plays a Crucial Role in Telomere Dysfunction‐Linked Atrial Remodeling

To further investigate the molecular mechanisms associated with atrial remodeling in F3 mice, we performed RNA‐Seq analysis on tissues isolated from the right atria of both WT and F3 mice. This analysis identified a total of 1267 differentially expressed genes, with 488 genes up‐regulated and 779 genes down‐regulated in F3 mice compared to WT mice.

**FIGURE 4:**
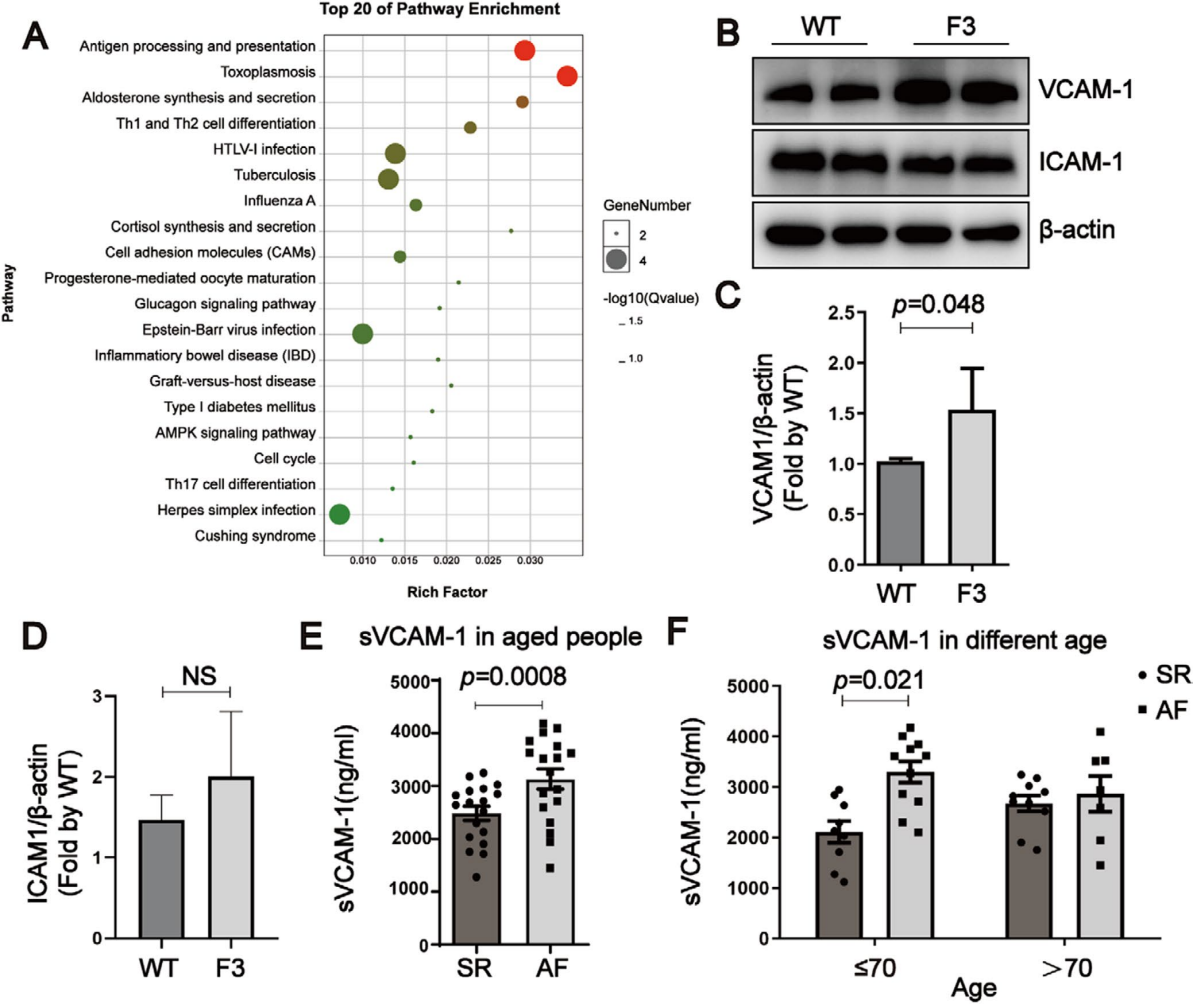
Potential role of adhesion molecules in atrial fibrillation induced by telomere shortening. (A) Scatter plot showing the results of KEGG enrichment analysis for differentially expressed genes in F3 mice compared to WT mice (*n* = 3). The top 20 significantly enriched pathways are highlighted, reflecting changes in adhesion molecule‐related pathways. (B) Western blot analysis of cell adhesion molecules VCAM‐1 and ICAM‐1 in WT and F3 mice, illustrating differences in expression levels associated with telomere shortening. (C, D) Quantification of VCAM‐1 and ICAM‐1 relative expression levels in WT and F3 mice, with β‐actin used as the loading control to ensure equal protein loading. (E) Comparison of serum VCAM‐1 concentrations between aged individuals with atrial fibrillation (AF) and those without AF, highlighting elevated levels in the AF group. (F) Analysis of serum VCAM‐1 concentrations across different age groups, demonstrating variations in VCAM‐1 levels with aging. Data are presented as mean ± SEM.

Enrichment analysis and Kyoto Encyclopedia of Genes and Genomes (KEGG) pathway analysis revealed significant alterations in pathways related to cell adhesion molecules (CAMs) (Figure 4A). In the subsequent protein expression analysis, VCAM‐1 was significantly elevated in the atrial tissues of F3 mice, while ICAM‐1 levels remained relatively unchanged (Figure 4B–D). Given the role of CAMs in cell adhesion, migration, and cellular signaling, the increased VCAM‐1 expression may provide insights into the mechanism of atrial remodeling in F3 mice.

The serum level of VCAM‐1 was then determined in clinical samples. The results showed that, in aged people (age ≥ 60 years), patients with AF (*n* = 18) exhibited significantly higher levels of serum free VCAM‐1 (sVCAM‐1) compared to individuals with normal sinus rhythm (*n* = 18) (Figure 4E). Subgroup analysis by age revealed that in young‐old (aged 60–70 years), sVCAM‐1 levels were significantly higher in those with AF (*n* = 12) compared to those in sinus rhythm (*n* = 10) (Figure 4F), suggesting that shortened telomere length contributes to increased sVCAM‐1 expression, thereby playing a role in AF induction. In contrast, in the old‐old group (aged over 70 years), no significant difference in sVCAM‐1 expression was observed between patients with AF (*n* = 6) and those with sinus rhythm (*n* = 8) (Figure 4F). This indicates that, while sVCAM‐1 plays a crucial role in telomere dysfunction‐linked atrial remodeling in young‐old (aged 60–70 years), in old‐old populations (aged over 70 years), other factors may overshadow the impact of shortened telomere length on AF, potentially influencing the altered sVCAM‐1 expression.

### 2.5 Reversal of Atrial Remodeling Through Anti‐VCAM‐1 Neutralizing Antibody in *TERT* ^−/−^ Mice

**FIGURE 5:**
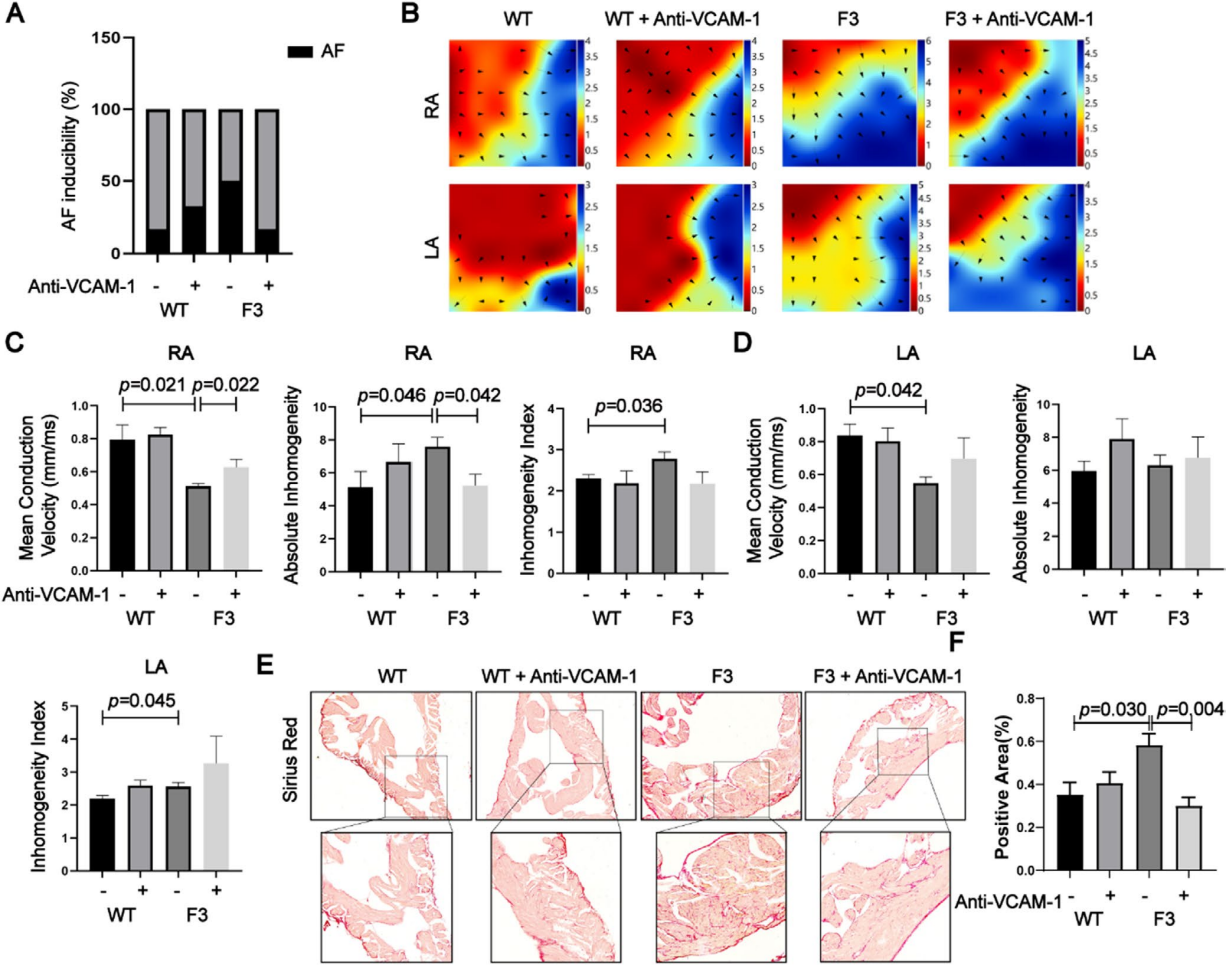
VCAM‐1 neutralizing antibody reduces atrial electrical and structural abnormalities, mitigating AF onset. (A) Comparison of atrial fibrillation inducibility in WT mice and F3 mice with and without VCAM‐1 neutralizing antibody treatment, showing a significant reduction in AF susceptibility in the treated F3 mice. (B) Simulated epicardial activation map illustrating changes in atrial electrical conduction in WT mice and F3 mice with and without VCAM‐1 neutralizing antibody treatment, with improved conduction patterns observed in the treated F3 mice. (C, D) Mean epicardial electrical conduction velocity, absolute inhomogeneity, and inhomogeneity index in different groups, highlighting significant improvements in conduction uniformity and velocity in the treated F3 group. (E) Sirius red staining of atrial tissue from in WT mice and F3 mice with and without VCAM‐1 neutralizing antibody treatment, demonstrating differences in fibrosis levels. (F) Quantification of sirius red staining in WT mice and F3 mice with and without VCAM‐1 neutralizing antibody treatment, showing a significant reduction in fibrosis in the treated F3 mice. Data are presented as mean ± SEM (*n* = 6).

To investigate the roles of VCAM‐1 in AF pathogenesis, we administered an anti–VCAM‐1 neutralizing antibody to both wild‐type (WT) and third‐generation *TERT* ^−/−^ (F3) mice. After 1 month of treatment, F3 mice receiving VCAM‐1 blockade exhibited a substantial reduction in AF inducibility. Specifically, the AF inducibility decreased by 30% compared to untreated F3 controls (Figure 5A; *n* = 6 per group), highlighting a critical contribution of VCAM‐1 to AF susceptibility in the context of telomere dysfunction.

To elucidate the underlying mechanisms, comprehensive mapping studies focusing on electrical conduction within the atria were conducted. We found F3 mice had a lower mean epicardial conduction velocity in both the right atrium and the left atrium compared with WT mice. Neutralizing antibodies effectively reversed the reduced electrical conduction velocity in the F3 mice right atrium and tended to alleviate left atrial electrical hyperconductivity compared with WT mice (Figure 5B–D). Notably, electrical inhomogeneity—quantified by absolute inhomogeneity and inhomogeneity index—was also improved, particularly in the right atrium, suggesting normalization of conduction patterns. This correction is crucial, as electrical inhomogeneity is a key factor in the initiation and maintenance of reentrant arrhythmias, which are fundamental to AF pathophysiology.

To evaluate structural remodeling, Sirius Red staining was employed to visualize atrial fibrosis. F3 mice treated with anti–VCAM‐1 antibody displayed markedly reduced collagen deposition in both atria compared to untreated F3 mice (Figure 5E–F). Atrial fibrosis, driven by excessive extracellular matrix (ECM) accumulation, disrupts normal tissue architecture and electrical conduction, thereby contributing to arrhythmogenesis. Our findings indicate that VCAM‐1 not only modulates electrical remodeling but also plays an essential role in fibrotic remodeling induced by telomere dysfunction.

### 2.6 VCAM‐1 Reverses Atrial Extracellular Matrix Changes in Telomere Dysfunctional Mice

**FIGURE 6:**
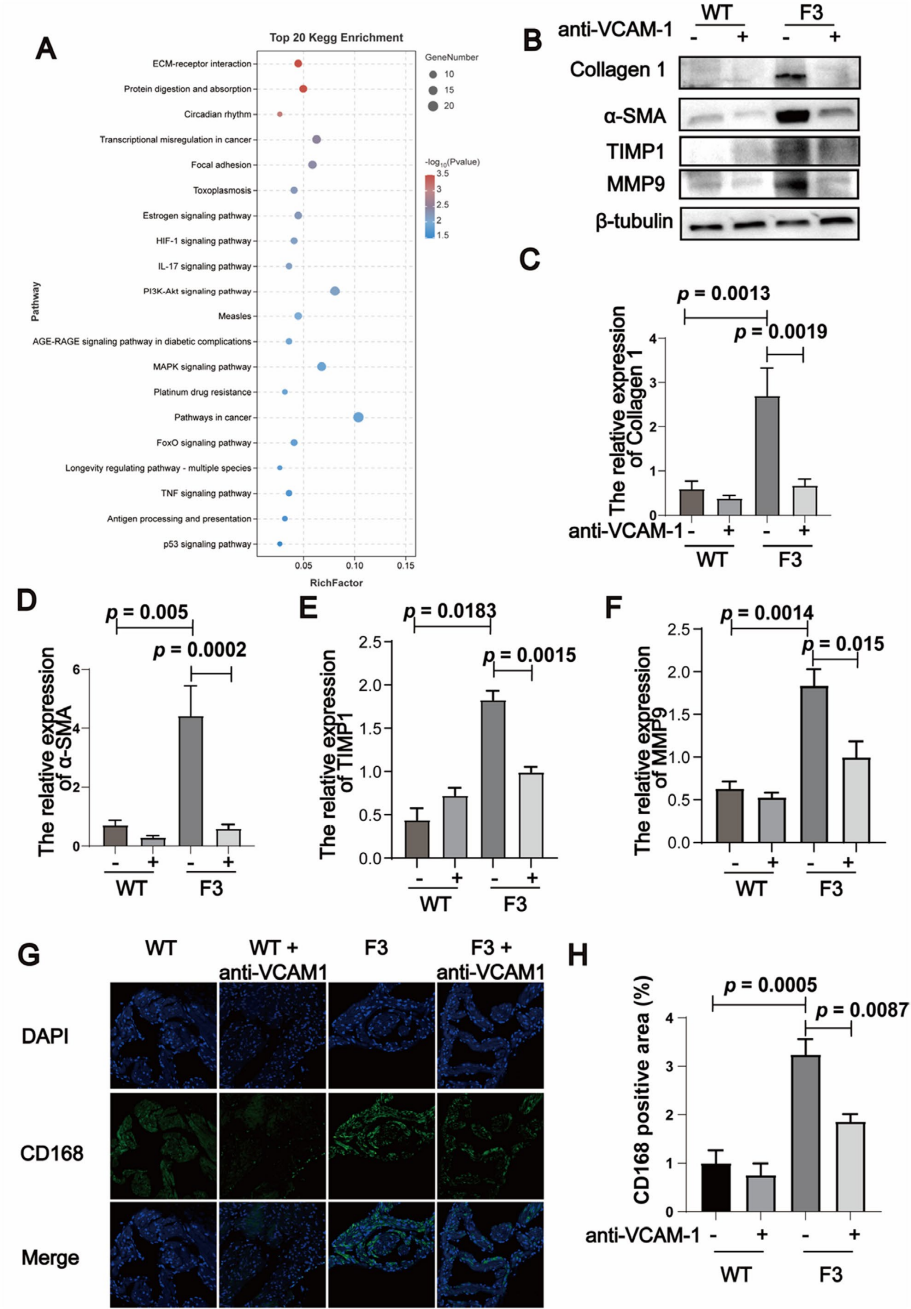
VCAM‐1 neutralizing antibody modulates extracellular matrix remodeling. (A) Scatter plot showing Kyoto Encyclopedia of Genes and Genomes (KEGG) pathway enrichment analysis of differentially expressed genes in F3 mice treated with VCAM‐1 neutralizing antibody compared to untreated F3 mice (*n* = 3). The top 20 significantly enriched pathways are displayed. (B) Western blot analysis of Col1α1, α‐SMA, TIMP1, and MMP9 expression in WT mice and F3 mice with and without VCAM‐1 neutralizing antibody treatment. β‐tubulin was used as a loading control. (C–F) Quantification of Col1α1, α‐SMA (*n* = 6), TIMP1 and MMP9 (*n* = 3) expression in different groups of mice as shown in B. (G) Immunofluorescence (IF) staining for CD168 in atrial tissue, where green fluorescence indicates CD168 expression. (H) Graph showing the proportion of CD168‐positive area in different groups of mice, reflecting changes in extracellular matrix remodeling. Data are presented as mean ± SEM.

To further understand the mechanism by which VCAM‐1 contributes to electrical and structural remodeling, we conducted RNA‐Seq analysis comparing untreated F3 mice with the VCAM‐1 neutralizing antibody treated mice. This analysis identified 1165 differentially expressed genes: 709 up‐regulated and 456 down‐regulated in the treated F3 mice. Enrichment and KEGG pathway analyses highlighted significant changes in pathways related to the ECM and its receptors, including collagens, integrin beta, and laminin gamma (Figure 6A).

We next assessed the expression levels of collagen‐1α and α‐SMA, which are among the most obvious molecules in RNA‐Seq analysis and also the key components of the ECM. In the atrial tissues of F3 mice, collagen‐1α and α‐SMA were significantly increased, while treatment with the VCAM‐1 neutralizing antibody reduced their expression (Figure 6B–F). CD168, also known as Hmmr, is among the most obvious molecules in RNA‐Seq analysis. It plays a crucial role in ECM remodeling by regulating matrix metalloproteinases (MMPs) and tissue inhibitors of metalloproteinases (TIMPs), which are vital for ECM turnover and are often dysregulated in fibrosis (Figure 6B–F). We observed a significant increase in CD168 expression in the atrial tissues of F3 mice compared to WT mice (Figure 6G,H), correlating with enhanced ECM remodeling and fibrosis. Treatment with the VCAM‐1 neutralizing antibody reduced CD168 levels to those comparable to WT mice (Figure 6G,H). These data demonstrate that F3 *TERT* ^−/−^ mice exhibit dysregulated MMP/TIMP balance consistent with enhanced ECM remodeling, and that VCAM‐1 neutralization partially rescues MMP expression and TIMP1 levels, in parallel with the reduction in CD168 expression, which provided experimental support linking CD168 and VCAM‐1 signaling to altered ECM turnover.

Collectively, these data demonstrate that VCAM‐1 is a key mediator of atrial electrical and structural remodeling in the setting of telomere dysfunction. Targeting VCAM‐1 not only reduced AF susceptibility but also reversed key electrophysiological and histological abnormalities associated with shortened telomeres. Our RNA‐seq analysis further corroborated these phenotypic improvements by revealing coordinated transcriptomic reprogramming of ECM‐related pathways (Figure 6A).

## 3 Discussion

Our study provides new insights into the interplay between aging, telomere dysfunction, and AF. Utilizing a novel quantitative dot blot technique, we discovered a significant association between LTL shortening and the increased susceptibility to AF in both murine models and human subjects. Our findings reveal that telomere dysfunction, particularly through the modulation of VCAM‐1, contributes to AF development by affecting ECM remodeling, leading to increased atrial fibrosis. Importantly, the use of anti‐VCAM‐1 antibodies effectively reduced AF inducibility and mitigated atrial remodeling in telomere‐shortened mice, underscoring the potential therapeutic value of targeting VCAM‐1 in preventing AF associated with telomere dysfunction.

Telomeres are well‐established markers of cellular aging and replicative capacity. Previous studies investigating the relationship between LTL and AF have often relied on telomere length measurement by PCR, whose accuracy is limited. Our quantitative dot blot method offers a high‐throughput solution with improved accuracy. The results from this clinical study suggest that telomere dysfunction and shortening may be important mechanisms underlying age‐related atrial fibrillation. To confirm this phenomenon, we generated *TERT* ^−/−^ mice, then assessed alterations in atrial electrical conduction using multi‐conductivity mapping. The results indicate that as telomeres shorten, the effective refractory period in mice decreases, and the inducibility of atrial fibrillation significantly increases.

To explore the mechanisms underlying AF induced by telomere dysfunction and shortening, we performed RNA‐Seq analysis on the atrial tissues of *TERT* ^−/−^ and WT mice. Previous studies have reported elevated VCAM‐1 levels in aging tissues, with associations between circulating levels of VCAM‐1 and AF incidence observed in clinical settings (Merat et al. 2000; Zhai et al. 2022; Mendez et al. 2021; Verdejo et al. 2011; Harling et al. 2017; Willeit et al. 2017; Zhai et al. 2022). Elevated levels of VCAM‐1 and ICAM‐1 have also been linked to reduced left atrial systolic function, suggesting that these adhesion molecules might exacerbate AF by impairing atrial function (Mathew et al. 2024). Our study is the first to establish a direct role of VCAM‐1 in promoting atrial remodeling and AF in *TERT* ^−/−^ mice, highlighting its importance as a molecular factor in age‐related AF. Furthermore, our research uniquely reports on the association between VCAM‐1 and aging‐related AF populations, adding a new dimension to the understanding of VCAM‐1's role in AF.

Previous research, including the Bruneck study, has established a link between elevated VCAM‐1 levels and AF occurrence (Willeit et al. 2017). Additionally, VCAM‐1 demonstrated significant diagnostic utility in postoperative AF, with a sensitivity of 60.0% and specificity of 77.3%, yielding an overall prediction accuracy of 75.2% (Harling et al. 2017). This underscores the potential of VCAM‐1 as a biological marker for AF. Notably, our study extends these findings by evaluating VCAM‐1's role as a biomarker specifically in the context of aging. Among young‐old individuals (60–70 years), AF patients showed significantly elevated plasma VCAM‐1 levels compared to sinus controls. In old‐old individuals (> 70 years), only an upward trend was observed. This parallels our LTL results, implying that AF in old‐old individuals may involve additional pathogenic mechanisms beyond telomere‐driven VCAM‐1 activation. The lack of statistically significant difference in VCAM‐1 expression compared to controls in the old‐old group (> 70 years) may be due to the complex interplay of multiple factors influencing AF in this population, extending beyond the effects of telomere shortening alone. Although the present study identifies VCAM‐1 as a key mediator linking telomere dysfunction to atrial remodeling and atrial fibrillation (AF) susceptibility, the upstream mechanisms driving VCAM‐1 upregulation warrant further consideration. Previous studies have shown that VCAM‐1 expression is tightly regulated by inflammatory cytokines, oxidative stress, and stress‐responsive signaling pathways, including NF‐κB, PI3K/AKT, and MAPK. In endothelial cells, pro‐inflammatory stimuli such as TNF‐α or lipopolysaccharide induce VCAM‐1 transcription through coordinated activation of these pathways, whereas inhibition of NF‐κB or MAPK signaling markedly suppresses VCAM‐1 expression (Pietersma et al. 1997; Trivedi and Jena 2013). In the context of telomere dysfunction, persistent telomere‐associated DNA damage activates the DNA damage response and promotes reactive oxygen species production and a chronic pro‐inflammatory milieu. We therefore propose that telomere shortening–induced genomic stress acts upstream of VCAM‐1 by engaging ROS‐ and cytokine‐driven NF‐κB/MAPK signaling, ultimately leading to VCAM‐1 upregulation and subsequent atrial remodeling. This framework provides a mechanistically plausible link between telomere attrition, inflammatory signaling, and VCAM‐1–dependent atrial remodeling in AF.

To further explore the key mechanisms by which VCAM‐1 promotes atrial remodeling and AF occurrence in telomere dysfunctional individuals, transcriptome sequencing analysis was conducted on *TERT* ^−/−^ mice with and without VCAM‐1 neutralizing antibodies. The results revealed a significant elevation in extracellular matrix‐related genes in *TERT* ^−/−^ mice, confirming the occurrence of atrial fibrosis. VCAM‐1 has been recognized as a key factor in atherosclerosis, and pharmacological strategies to selectively inhibit VCAM‐1 function have emerged in recent years, particularly peptide‐ and antibody‐based novel therapies (Sano et al. 2021; Pickett et al. 2023). However, research on these VCAM‐1‐directed therapies remains scarce (Pickett et al. 2023). The novelty of our study lies in the use of anti‐VCAM‐1 neutralizing antibodies, which can prevent atrial remodeling, reverse ECM changes (Col1α1 and α‐SMA), reduce fibrosis, and prevent AF induction.

Furthermore, CAMs are pivotal in the progression of fibrosis across various organs, including the lungs, kidneys, skin, and liver. These molecules facilitate cell–cell and cell‐ECM interactions, essential for maintaining tissue structure and homeostasis. Aberrant expression of CAMs has been observed during different stages of fibrosis, including acute and chronic inflammation, fibroblast activation, epithelial‐mesenchymal transition (EMT), and ECM remodeling. CAMs also modulate fibrotic signaling pathways, such as the Wnt/β‐catenin and TGF‐β pathways, which are well‐known contributors to fibrosis (Hu et al. 2023). In liver fibrosis, CAMs play a dual role by serving as adhesion structures and signal transducers, facilitating cell‐ECM binding and cell–cell adhesion. Furthermore, CAMs are involved in complex interactions that can broadly promote fibrosis, including the release of pro‐fibrotic TGF‐β and mediation of leukocyte migration. Given their integral role in fibrosis, several CAMs have been investigated as therapeutic targets for preventing or reversing liver inflammation and fibrosis (Hintermann and Christen 2019). Collectively, these findings underscore the importance of CAMs, particularly VCAM‐1, in regulating the extracellular matrix and contributing to fibrotic processes. Collectively, our findings underscore the importance of CAMs, particularly VCAM‐1, in regulating the extracellular matrix and contributing to fibrotic processes, ultimately leading to structural and functional remodeling of the atria.

An important mechanistic question raised by our findings is how VCAM‐1 signaling promotes extracellular matrix (ECM) remodeling. CD168 (HMMR), identified as a prominently altered gene in our RNA‐seq analysis, has been shown to enhance matrix metalloproteinase (MMP) expression through NF‐κB and MAPK signaling at inflammatory sites. In line with this, telomere‐dysfunctional atrial tissue exhibited coordinated upregulation of CD168 together with dysregulation of MMPs and TIMP1, consistent with pathological ECM remodeling. Importantly, VCAM‐1 neutralizing antibody attenuated CD168 expression and partially restored MMP/TIMP balance, supporting a model in which VCAM‐1 acts upstream of a CD168‐MMP to drive atrial structural remodeling and increase susceptibility to atrial fibrillation.

In summary, our findings provide new insights into the role of telomere dysfunction and VCAM‐1 in the pathogenesis of AF. These results highlight the potential of VCAM‐1 as a therapeutic target for preventing and treating AF, particularly in the context of aging. Further studies are warranted to explore the clinical applicability of these findings and to develop targeted therapies for AF based on telomere biology and VCAM‐1 modulation.

## 4 Methods and Materials

### 4.1 Quantitative Dot Blot Assay

Whole blood samples were collected from individuals with AF or sinus rhythm. DNA was extracted using the Whole Blood Genomic DNA Extraction Kit (WEGO, China), ensuring that the samples were homologous to the test samples. 30 ng of extracted DNA was taken and added to 100 μL of 0.1 M NaOH. The mixture was incubated at 37°C for 15 min, then an equal volume of 2 × SSC (0.3 M NaCl, 30 mM Na_3_C_6_H_5_O_7_ × 2H_2_O) was added and mixed thoroughly. The filter paper and nylon membrane were prepared by immersing them in 70% ethanol for 1 min, followed by four washes with 2 × SSC, each lasting 5 min. The BIO‐DOT apparatus was assembled, and sequential dot blotting was performed. Once blotting was complete, the nylon membrane was fixed using a multifunctional crosslinker set at an energy of 1.2 × 10^5^ μJ/cm^2^. The membrane was prehybridized at 60°C for 45 min, then hybridized with the β‐actin probe at 60°C for 9 h. The membrane was washed with Washing Buffer I (2 × SSC + 0.1% SDS) at room temperature twice for 5 min each. This was followed by two washes using Washing Buffer II (0.2 × SSC + 0.1% SDS) at 68°C, each lasting 15 min. The membrane was washed with Washing Buffer (0.1 M maleic acid, 0.15 M NaCl, 0.3% Tween‐20) at room temperature for 5 min. 10 mL of 1× blocking solution was added, and incubation was performed at room temperature for 45 min. The anti‐DIG‐AP antibody (Switzerland, Roche) was applied at a 1:4000 dilution and incubated at room temperature for 3 h. The membrane was washed twice with Washing Buffer at room temperature for 15 min each, followed by a single wash with 1× detection buffer (0.1 M NaCl, 0.1 M Tris HCl) at room temperature for 15 min. Finally, the membrane was incubated with an appropriate amount of CD Star Ready‐to‐use coloration solution (Roche, Switzerland) at room temperature for 15 min and exposure was performed using a chemiluminescence imager.

### 4.2 Population Inclusion Criteria and Follow‐Up

This study was approved by the Ethics Committee of the Second Hospital of Tianjin Medical University and has been registered with the Chinese Clinical Trial Registry (Registration number: ChiCTR2200056733) and adhered to the Declaration of Helsinki. This was a single‐center, prospective cohort study that enrolled AF patients undergoing cryoablation treatment at the Second Hospital of Tianjin Medical University from July 2020 to July 2021. Participants in the sinus rhythm group consisted of patients with sinus rhythm who were admitted to the Tianjin Workers Hospital during the same period.

The inclusion criteria were as follows: (1) First‐time cryoballoon ablation for AF; (2) Age ≥ 18 years; (3) Transesophageal echocardiography (TEE) ruled out thrombus in the left atrium and left atrial appendage; (4) Agreement for regular ECG or Holter monitoring every 3 months post‐ablation is required. Exclusion criteria included: (1) Prior history of left atrial ablation or cardiac surgery (e.g., left atrial appendage occlusion); (2) Life expectancy of < 1 year; (3) Poor patient compliance, leading to missed follow‐ups. Baseline information for these patients has been provided in Tables S1 and S2. Categorical variables are presented with *n* values and percentages. Continuous variables are presented with median and interquartiles.

Blood samples (5 mL) were collected from the vein prior to cryoablation in the AF group and upon admission for the non‐AF group. The samples were placed into ethylenediaminetetraacetic acid (EDTA) anticoagulation tubes, centrifuged at 800×*g* for 20 min at 4°C, aliquoted into cryovials, and stored at −80°C until biomarker analysis. Follow‐up appointments were scheduled for patients in the AF group at 3, 6, 9, and 12 months post‐ablation. Patients received anticoagulant therapy for at least 2 months post‐ablation, with anti‐arrhythmic drugs permissible during the three‐month blanking period post‐ablation. The primary endpoint was the recurrence of atrial arrhythmias within the 90‐day blanking period post‐ablation, defined as episodes of AF, atrial flutter (AFL), or atrial tachycardia (AT) lasting at least 30 s on ECG or Holter monitoring.

### 4.3 UK Biobank‐Based Bioinformatic Analysis

To investigate aging‐related differences in leukocyte telomere length (LTL) and the risk of AF, we conducted a parallel bioinformatic analysis using data from the UK Biobank (https://www.ukbiobank.ac.uk/), a large‐scale, prospective, population‐based cohort study comprising over 500,000 participants aged 37–73 years at recruitment between 2006 and 2010. Participants with missing data or those who responded “prefer not to answer” or “don't know” to any covariates or LTL‐related variables were excluded. After quality control, a total of 431,445 participants were included in the final analysis.

Leukocyte telomere length (UK Biobank field ID: 22192) was log‐transformed and *z*‐standardized due to its non‐normal distribution and treated as a continuous variable. The definition of AF (including atrial flutter) is provided in Table S1. Multivariable‐adjusted Cox models were used to evaluate the association between LTL and incident AF. To illustrate cumulative risk over time, multivariable‐adjusted Kaplan–Meier survival curves were constructed based on mean LTL levels, with adjustment using inverse probability weighting.

A stratified analysis was further conducted by age group (< 70 vs. ≥ 70 years). All models were adjusted for potential confounders, including age, sex, race, smoking status, body mass index (BMI), systolic and diastolic blood pressure, use of antihypertensive medications, and presence of diabetes. Statistical significance was defined as a two‐sided *p*‐value < 0.05. All statistical analyses were conducted using R version 4.2.3 (R Foundation for Statistical Computing).

### 4.4 Southern Blot

DNA was extracted from human blood, and the concentration was measured. Then, 500 ng of DNA was added to a 1% agarose gel for electrophoresis under the following conditions: 4°C, U = 100 V, 3 h. After electrophoresis, the gel was transferred to a nylon membrane using a BIO‐DOT apparatus, and the subsequent steps were performed similarly to a quantitative dot blot assay.

### 4.5 Animals and Antibody Treatment

*TERT* ^*+/−*^ mice were a gift from Professor Yusheng Cong of Hangzhou Normal University; the genetic background of the *TERT* ^*+/−*^ mice is C57BL/6J. To generate *TERT* ^*−/−*^ mice, we performed crossbreeding using heterozygous *TERT* ^*+/−*^ mice. WT mice used in this study were generated as offspring of heterozygous (*TERT* ^*+/−*^) breeding pairs. All mice were housed in a specific pathogen‐free environment, provided with full‐price diet, and had access to autoclaved water. Each cage housed no more than five mice. At 10 weeks of age, WT and F3 mice received intraperitoneal injections of saline or VCAM‐1 neutralizing antibody (9 mg/kg) every 3 days for a total of nine injections. Following the injections, in vivo endocardial electrophysiological experiments and epicardial electrogram recordings were conducted. Thereafter, the heart was promptly excised at the root of the aorta, rinsed in pre‐cooled 4°C 1 × PBS to remove residual blood, and then air‐dried on a cold plate using filter paper. Excess fat and connective tissue were removed, and the entire heart, atrium, and ventricle were weighed using an electronic scale. Following weighing, a portion of the left atrial tissue was fixed in a 4% paraformaldehyde solution for subsequent pathological experiments. The remaining left atrial tissue was immediately frozen in liquid nitrogen. All experiments were approved by the Animal Ethics Committee of the Tianjin Medical University (TMUaMEC2022015).

### 4.6 Electrophysiology Experiment

The mice were anesthetized with 1.5% tribromoethanol (20 mg/kg). Prior to the surgery, a standard lead II surface electrocardiogram (ECG) was recorded for 5 min. The sinus cycle length (SCL) was initially recorded, and then pacing at twice the threshold value was performed to determine the pacing threshold. The atrial effective refractory period (AERP) involved issuing a premature stimulus S2 after every eight consecutive basic pacing S1, with S2 decrementing in 2 ms steps until the longest S1S2 interval at which the atrium could no longer be captured. AF induction was performed using burst stimulation, with a frequency of 40 ms–20 ms (duration of a single stimulus), decreasing by 2 ms, and continuous S1S1 stimulation for 30 s (no interval between two single stimuli), with a 1‐min interval, repeated five times. The number of AF episodes and their duration were recorded. AF was defined as rapid, irregular atrial activations on the ECG following burst stimulation, lasting ≥ 1 s. The formula for calculating the incidence rate of AF is: number of mice exhibiting AF (mice)/Total number of mice (mice) × 100%.

### 4.7 In Vivo Epicardial Electromapping

Following the completion of the in vivo endocardial electrophysiological experiment, the trachea was intubated and connected to a small animal ventilator. A thoracotomy was performed along the midline. Epicardial mapping experiments were conducted using the EMapScope3.0 operating system (MappingLab, UK). A multi‐array microelectrode mapping system consisting of 36 electrodes (6 × 6) was placed on the atrial epicardium for electrical conduction mapping, specifically targeting the left atrium. Upon completion of the mapping, the EMapScope4.0 analysis software was used to calculate the CV. Heterogeneity was measured using the absolute inhomogeneity index, as described previously in detail.

### 4.8 Sirius Red Staining

Stain with the Sirius Red staining kit (Solarbio, China). Briefly, the processed atrial tissue sections were placed on glass slides. The sections were re‐dehydrated with 70% ethanol for approximately 5 min. The sections were stained in an iron hematoxylin staining solution for 5 min, followed by a 15 s rinse with distilled water to remove excess stain. The slides were then immersed in Sirius Red staining solution for approximately 15 min, followed by a water rinse to remove excess stain. The sections were dehydrated through a series of gradually increasing ethanol concentrations (75%, 95%, 100% ethanol) and finally cleared in xylene before being mounted with neutral balsam. The degree of interstitial fibrosis in atrial tissue was determined by calculating the percentage of the red‐stained area to the total tissue area in the images (excluding the fibrotic areas around blood vessels). We employed the ImageJ image analysis software to quantify the area of Sirius Red‐positive staining within the tissue. Concurrently, the total atrial tissue area within each field of view was measured using the same software. The ratio of Sirius Red‐positive staining was calculated by dividing the stained area by the total atrial tissue area in the corresponding field of view, providing a quantitative assessment of collagen deposition in the atrial tissue. For clarity and consistency, all Sirius Red‐positive staining areas reported in this study are presented as the proportion of staining within the atrial tissue of the respective field of view.

### 4.9 Telomere DNA FISH

Mice were weighed, which was followed by colchicine injection (MedChemExpress, USA) intraperitoneally at a dose of 40 μg per gram of body weight. After 16 h, euthanize the mice, remove the limbs, shave off the muscles, and cut off the joints at both ends. A syringe containing 0.075 M KCl was inserted into one end of the bone. The bone marrow cells were then flushed into a clean centrifuge tube. After low permeation at 37°C for 40 min, 5 drops of pre‐cooled fixative (methanol: acetic acid = 3:1) were added and fixation was performed for 5 min. Centrifugation was performed at 900 rpm for 9 min, following which the supernatant was discarded. 10 mL of fixative was added while shaking the centrifuge tube. The sample was left at room temperature for 30 min, re‐centrifuged at 900 rpm for 9 min. The supernatant was then discarded; 1 mL of fixative was added to resuspend the cells. Slides were then made and placed in a staining dish. They were washed twice with 1 × PBS each time for 5 min. 2% paraformaldehyde (Solarbio, China) was added for fixation for 20 min, followed by washing three times with 1 × PBS for 5 min each. Pepsin (Sigma, USA) was added and the sample was left at 37°C for 5 min, and then washed twice with 1 × PBS for 5 min each. The slides were taken out and air dried. Telomere probe hybridization solution (70% formamide (Sigma, USA), 10 mM Tris–HCl (pH = 7.2), 2.5 ng/mL TeloC‐488 (Panagene, South Korea)) was added for hybridization at 80°C for 3 min, and then incubated in the dark at room temperature for 2 h. Washing was performed vigorously twice with wash buffer I (70% formamide, 0.1% BSA, 10 mM Tris–HCl (pH = 7.2)), each time for 15 min. TBST (0.15 M NaCl, 0.5 M Tris–HCl, 0.1% Tween‐20) was further used to wash the samples three times for 5 min each. After air‐drying the slides, they were sealed with DAPI, and fluorescence microscopy was used for visualization (Nikon, Japan).

### 4.10 Immunofluorescence With Fluorescent In Situ Hybridization (IF‐FISH)

Bone marrow cells were placed on a coverslip on a 6‐well plate. The α‐MEM medium (Corning, USA) was discarded, and the remaining sample was washed once with 1 × PBS for 5 min. 2% paraformaldehyde was added for fixation for 20 min, washed twice with 1 × PBS, each time for 5 min. Permeabilization was achieved using 0.5% TritonX‐100 in 1 × PBS for 20 min, followed by two washes with 1 × PBS, each for 5 min. The coverslip was removed, the sample was air dried, and then hybridized using the telomere probe (TeloC‐Cy3). After completion, block with a solution containing 1% BSA and Galetine in 1 × PBS for 1 h. Subsequently, incubation with γH2AX antibody for 1 h was performed, followed by washing four times with PBST (1 × PBS containing 0.1% Tween‐20), each time for 5 min. The sample was then incubated with fluorescent secondary antibody at room temperature in the dark for 30 min, followed by four washes with PBST, each for 5 min. After drying, mount the coverslip with DAPI, and fluorescence microscopy was used for visualization.

### 4.11 Immunofluorescence (IF)

The paraffin sections were deparaffinized in xylene for 5 min, followed by a second deparaffinization in fresh xylene for 5 min, repeated twice. Subsequently, the sections were treated with absolute ethanol for 5 min, twice, followed by 90% ethanol for 5 min, twice, and 70% ethanol for 5 min. After these ethanol treatments, the sections were rinsed with distilled water for 5 min, repeated twice. The slides were then immersed in 1× sodium citrate antigen retrieval solution (Beyotime, China) and heated at 95°C for 20 min before cooling to room temperature. After washing three times with 1 × PBS for 5 min each, the sections were incubated with 5% BSA for 30 min. Following this, the primary antibody was applied and incubated at 37°C for 1 h. The sections were rinsed three times with distilled water for 5 min each. Subsequently, the fluorescence secondary antibody was incubated at 37°C for 2 h, followed by three washes with distilled water, each for 5 min. After covering the specimens with DAPI and allowing it to act for 5 min at room temperature, the slides were rinsed three times with distilled water for 5 min each before being sealed for observation.

### 4.12 RNA Extraction and Sequencing (RNA‐Seq)

The main steps of mRNA acquisition are presented as follows. Firstly, using the structural feature that most of the eukaryotic mRNAs have polyA tails, mRNAs with polyA tails are enriched by Oligo (dT) magnetic beads. mRNAs obtained are subsequently randomly interrupted with divalent cations in NEB Fragmentation Buffer and constructed according to the NEB common library construction method or strand‐specific library construction method [2]. NEB normal library construction: the first strand of cDNA was synthesized in M‐MuLV reverse transcriptase system using the fragmented mRNA as template and random oligonucleotides as primers, followed by degradation of the RNA strand with RNaseH, and synthesis of the second strand of cDNA with dNTPs in a DNA polymerase I system. After purified double‐stranded cDNA was end‐repaired, A‐tailed and connected to sequencing junction, cDNA around 250–300 bp was screened with AMPure XP beads, PCR amplification was performed and PCR products were purified again using AMPure XP beads to finally obtain the library. The library construction kit is NEBNext Ultra RNA Library Prep Kit for Illumina.RNA samples with an integrity (RIN) > 7 and 28S/18S ratio ≥ 1.8 were selected for subsequent sequencing. The extracted RNA was reverse transcribed into cDNA, and cDNA libraries were constructed and sequenced using the BGISEQ‐500 platform. Quality control and filtering were applied to the obtained sequencing reads. Differential gene analysis software, DESeq2, was utilized to determine the expression levels of genes by comparing the number of reads for each gene. Multiple testing (False Discovery Rate, FDR) was used to adjust *p* value, and *Q*‐value of ≤ 0.05 is considered statistically significant. KEGG was performed for pathway enrichment analysis of differentially expressed genes.

### 4.13 Western Blot

The frozen left atrial tissue was ground into powder under liquid nitrogen and then added to RIPA lysis buffer containing 1 mM PMSF for thorough mixing. The mixture was lysed on ice for 30 min, followed by centrifugation at 12,000*g* for 20 min at 4°C, and the supernatant was collected. 5 × SDS loading buffer was added to the supernatant, and the proteins were denatured at 100°C for 10 min. Denatured proteins were separated by 10% SDS‐PAGE and then transferred onto a PVDF membrane. After overnight incubation with the primary antibody, the membrane was washed three times with TBST for 15 min each. Subsequently, the membrane was incubated with the secondary antibody for 1 h, followed by TBST washing, and the immune reaction was detected through chemiluminescence.

### 4.14 Antibodies

Anti‐CD168 (Immunoway, YT5887), anti‐VCAM‐1 (BioXcell, BE0027; Abcam, ab134047), rat IgG1 isotype control (BioXcell, BE0088), anti‐α‐SMA (Abcam, ab5694), anti‐Col1A1 (91144s, Cell Signaling Technology), anti‐ICAM (Santa Cruz Biotechnology, sc‐107), anti‐β‐actin (Immunoway, YM3028), anti‐β‐tubulin (Immunoway, YM3030), anti‐γH2AX (Millipore, SAB5700699).

### 4.15 Statistical Analysis

GraphPad software was utilized for statistical analysis. Results are presented as mean ± standard error (SEM). Inter‐group differences were analyzed using the unpaired *t*‐test, with *p* < 0.05 considered statistically significant.

## Author Contributions

Zhaojia Wang was responsible for experimental design, animal model construction, electrophysiological experiments, molecular biology experiments, experimental statistics, and article writing. Rui Zhao handled the high‐throughput, single‐gene‐calibrated dot blot assay, as well as the breeding, identification of telomere knockout mice, and the detection and statistical analysis of leukocyte telomere length, with assistance from Duo Jiang in the telomere length detection and statistics. Yuwen Wang and Qiuhui Yang fed the telomere knockout mice, contributed to model establishment, and detected MMPs and TIMPs expression. Nan Zhang contributed to electrophysiological experiments of older *TERT* ^−/−^ mice. Zandong Zhou partially contributed to electrophysiological experiments, molecular biology experiments, and statistical analysis of some experimental data. Xu Zhang performed model establishment and minor electrophysiological experiments. Jinghua Yuan was responsible for the breeding and identification of telomere knockout mice. Yi Zheng participated in article revision. Wenhua Song managed clinical data collection and statistics. Daiqi Liu performed partial free VCAM‐1 level detection. Xunzhi Liu and Kejing Yuan provided partial technical support. Gary Tse and Gregory Y.H. Lip contributed to article revision and suggestions. Tong Liu oversaw the overall research design and article revision. Feng Wang directed the overall research design, article writing, and revision.

## Funding

This work was supported by the National Natural Science Foundation of China (32170762, 82170327, 82370332, 31771520, 92149302, 82072331, 82271265), Scientific Research Program of Tianjin Municipal Education Commission 2020KJ200 and the Tianjin Health Research Project (19YFZCSY00600), the Tianjin Key Medical Discipline (Specialty) Construction Project (TJWJ2022XK013, TJYXZDXK‐029A), Tianjin Natural Science Grant (19JCJQJC63500), the Natural Science Foundation of Tianjin city (23JCZDJC00270, 24JCQNJC00820, 25JCZDJC00510), the Non‐profit Central Research Institute Fund of Chinese Academy of Medical Sciences (2025‐JKCS‐23), CAMS innovation Fund for Medical Sciences (2021‐I2M‐1‐042, 2022‐I2M‐2‐003, 2022‐I2M‐2‐008), and Scientific Research Program of Tianjin Municipal Education Commission (2023KJ038).

## Conflicts of Interest

The authors declare no conflicts of interest.

## Supporting information


**Figure S1:** Lack of association between telomere length and AF recurrence. (A) Scatter plot showing the correlation between telomere length and the recurrence of atrial fibrillation (AF) in patients, illustrating no significant association between these two variables.
**Figure S2:** Telomere length shortening in TERT knockout F3 mice.
(A) Representative telomere Q‐FISH images of bone marrow cells from WT and F3 mice. Chromosomes are labeled with DAPI (blue), and telomeres are visualized with telomere‐specific PNA probes (green). (B) Histogram showing the distribution of relative telomere length in WT and F3 mice, measured as fluorescence intensity (TFU, telomere fluorescence unit) by Q‐FISH analysis. Green lines mean indicated mean TFU. Mean ± SEM of TFU was shown above each panel.
**Table S1:** Definition of AF in UK Biobank‐based bioinformatic analysis.

## Data Availability

The data that support the findings of this study are available on request from the corresponding author. The data are not publicly available due to privacy or ethical restrictions.
