# Supplementary material for: Telomere Shortening Drives Atrial Fibrillation Through VCAM‐1 Mediated Atrial Electrical and Structural Remodeling
Source: Aging Cell. 2026 Feb 13;25(2):e70417. doi: 10.1111/acel.70417 (PMC12903549; doi:10.1111/acel.70417)
Supplement: Supplementary file 1 — Figure S1: Lack of association between telomere length and AF recurrence. (A) Scatter plot showing the correlation between telomere length and the recurrence of atrial fibrillation (AF) in patients, illustrating no significant association between these two variables. Figure S2: Telomere length shortening in TERT knockout F3 mice. (A) Representative telomere Q‐FISH images of bone marrow cells from WT and F3 mice. Chromosomes are labeled with DAPI (blue), and telomeres are visualized with telomere‐specific PNA probes (green). (B) Histogram showing the distribution of relative telomere length in WT and F3 mice, measured as fluorescence intensity (TFU, telomere fluorescence unit) by Q‐FISH analysis. Green lines mean indicated mean TFU. Mean ± SEM of TFU was shown above each panel. Table S1: Definition of AF in UK Biobank‐based bioinformatic analysis. [file ACEL-25-e70417-s001.docx]

**Figure S1**


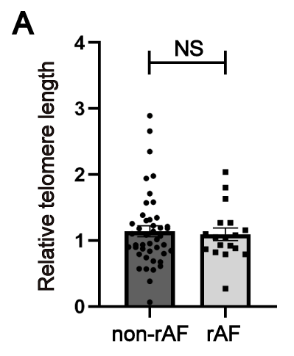


**Figure S1. Lack of association between telomere length and AF recurrence**

A. Scatter plot showing the correlation between telomere length and the recurrence of atrial fibrillation (AF) in patients, illustrating no significant association between these two variables.

**Figure S2**


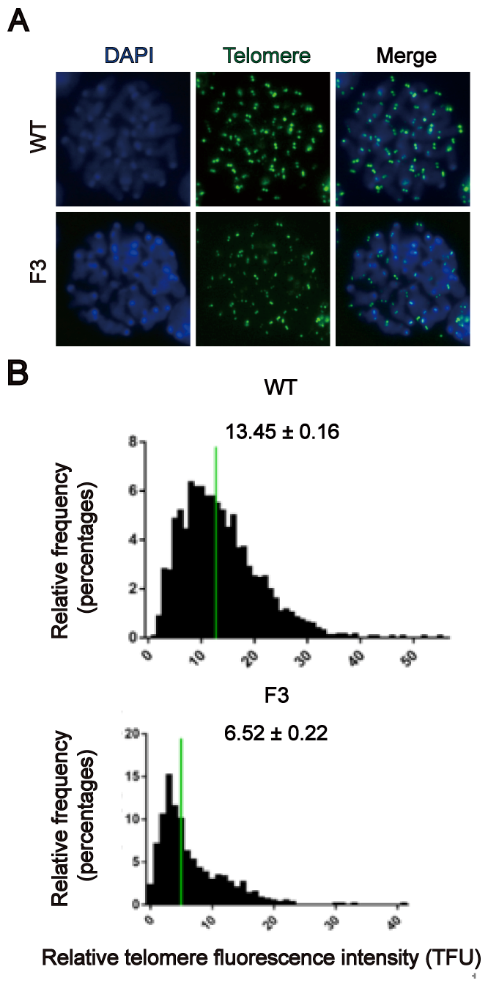


**Figure S2. Telomere length shortening in TERT knockout F3 mice**

A. Representative telomere Q-FISH images of bone marrow cells from WT and F3 mice. Chromosomes are labeled with DAPI (blue), and telomeres are visualized with telomere-specific PNA probes (green).

B. Histogram showing the distribution of relative telomere length in WT and F3 mice, measured as fluorescence intensity (TFU, telomere fluorescence unit) by Q-FISH analysis. Green lines mean indicated mean TFU. Mean ± SEM of TFU was shown above each panel.

**Table S1. Definition of AF in UK Biobank-based bioinformatic analysis**

|  | Field Name | Code | Definition |
| --- | --- | --- | --- |
| Atrial Fibrillation/Flutter | Diagnosis – ICD10 | I48, I48.1, I48.2, I48.3, I48.4, I48.9 | Atrial fibrillation and flutter; paroxysmal atrial fibrillation; persistent atrial fibrillation; chronic atrial fibrillation; typical atrial flutter; atypical atrial flutter; atrial fibrillation and atrial flutter, unspecified. |
|  | Underlying (primary) cause of death - ICD10 |  |  |
|  | Operative procedures - OPCS4 | K62.1, K62.2, K62.3, K62.4 | Percutaneous transluminal ablation of pulmonary vein to left atrium conducting system; Percutaneous transluminal ablation of atrial wall for atrial flutter; Percutaneous transluminal ablation of conducting system of heart for atrial flutter NEC; Percutaneous transluminal internal cardioversion NEC |
